# Use of hospital services by age and comorbidity after an index heart failure admission in England: an observational study

**DOI:** 10.1136/bmjopen-2015-010669

**Published:** 2016-06-09

**Authors:** Alex Bottle, Rosalind Goudie, Derek Bell, Paul Aylin, Martin R Cowie

**Affiliations:** 1 Dr Foster Unit at Imperial College, London, UK; 2 Chelsea and Westminster Hospital, Imperial College London, London, UK; 3 National Heart and Lung Institute, Imperial College London (Royal Brompton Hospital), London, UK

**Keywords:** EPIDEMIOLOGY

## Abstract

**Objectives:**

To describe hospital inpatient, emergency department (ED) and outpatient department (OPD) activity for patients in the year following their first emergency admission for heart failure (HF). To assess the proportion receiving specialist assessment within 2 weeks of hospital discharge, as now recommended by guidelines.

**Design:**

Observational study of national administrative data.

**Setting:**

All acute NHS hospitals in England.

**Participants:**

82 241 patients with an index emergency admission between April 2009 and March 2011 with a primary diagnosis of HF.

**Main outcome measures:**

Cardiology OPD appointment within 2 weeks and within a year of discharge from the index admission; emergency department (ED) and inpatient use within a year.

**Results:**

15.1% died during the admission. Of the 69 848 survivors, 19.7% were readmitted within 30 days and half within a year, the majority for non-HF diagnoses. 6.7% returned to the ED within a week of discharge, of whom the majority (77.6%) were admitted. The two most common OPD specialties during the year were cardiology (24.7% of the total appointments) and anticoagulant services (12.5%). Although half of all patients had a cardiology appointment within a year, the proportion within the recommended 2 weeks of discharge was just 6.8% overall and varied by age, from 2.4% in those aged 90+ to 19.6% in those aged 18–45 (p<0.0001); appointments in other specialties made up only some of the shortfall. More comorbidity at any age was associated with higher rates of cardiology OPD follow-up.

**Conclusions:**

Patients with HF are high users of hospital services. Postdischarge cardiology OPD follow-up rates fell well below current National Institute for Health and Care Excellence guidelines, particularly for the elderly and those with less comorbidity.

**Strengths and limitations of this study**

- Patients with heart failure (HF) frequently have high unplanned admission and readmission rates, but much less is known about their use of emergency departments and outpatient departments (OPDs) and the role of non-cardiology specialties. We made use of national administrative data for England that capture this activity.
- Linkage to death registrations and the use of cumulative incidence rates allowed OPD utilisation for cardiology and other specialties to be correctly calculated.
- We did not have data on subsequent follow-up in the community.

## Introduction

Heart failure (HF) is a serious chronic disease that is common in most countries. In the UK, it affects around 900 000 people with an estimated cost to the NHS of 1–2% of the annual budget.^1^ Responding to the limited knowledge on the epidemiology, clinical characteristics and outcomes of real-world patients with HF, the European Society of Cardiology (ESC) Heart Failure Registry was established, covering over 100 centres in 12 European countries other than the UK. Its pilot study reported wide differences in patient characteristics, treatment and outcomes for inpatients and outpatients.^2^ The annual national HF audits for England and Wales^3^ have also documented variations in care processes and outcomes. The sixth and most recent published national audit (2012–2013) shows for the first time a fall in mortality among contributing hospitals, consistent with international trends.^4^ Following hospitalisation, the challenge is to ensure a seamless transition from inpatient to outpatient care and integration with chronic HF management. The ESC guidelines recommend multidisciplinary management programmes with structured follow-up that includes patient education, optimisation of medical treatment, psychosocial support and improved access to care.^5^ Accordingly, there is a growing global focus on the timing of specialist follow-up as part of this transition. A Medicare and Get With The Guidelines study in the USA found that hospitals with the lowest rates of follow-up within 7 days of discharge had the highest 30-day readmission rates.^6^ The AHA guidelines describe a postdischarge follow-up visit within 7–14 days and/or a telephone follow-up within 3 days of hospital discharge as ‘reasonable’.^7^ The National Institute for Health and Care Excellence (NICE) guideline on diagnosing and managing acute HF in adults^8^ states that “a follow-up clinical assessment should be undertaken by a member of the specialist heart failure team within two weeks of the person being discharged from hospital.” We determined the proportion of patients offered a cardiology outpatient department (OPD) appointment within 2 weeks of discharge as a proxy for this and investigated how it varied by age and comorbidity.

Previous work has focused on aggregate emergency admission rates or on patient factors and hospital factors that predict readmission and mortality. These outcomes are important, but to better understand true demand a broader understanding of the use of other hospital services by HF patients is required. The NHS in England benefits from national linked data that encompass inpatient, day case, OPD and emergency department (ED) activity. To date there has been little published on data by HF patients in England or elsewhere. We describe this use in the year after an index HF admission, overall and related to age and comorbidity.

## Methods

### Data source

Hospital Episodes Statistics (HES) is the national administrative database for England and covers all NHS hospitals and Independent Sector Treatment Centres, totalling around 15 million records each year; similar systems exist for the other UK countries. Since 2003–2004, it has included OPD records (60 million records each year), and since 2007–2008 it has included ED records (now around 19 million each year). Records can be matched for the same patient using an identifier that uses a combination of unique NHS number, date of birth, sex, postcode and hospital number. Inpatient diagnosis fields use ICD10. Procedures are coded using the UK's own OPCS system.^9^ As the ED and OPD diagnosis fields are too infrequently populated to be useful, we restricted analyses of the ED portion of HES to the fact, date and outcome of the attendance and the OPD portion to the fact, date and specialty of the appointment.

### Patient cohort and subgroups

We extracted emergency admissions for HF (ICD10 I50 as the primary diagnosis) with discharge dates between April 2009 and March 2011: for each patient, the first of these admissions was defined as their index admission. Patients were excluded if they had had an emergency admission with a primary diagnosis of HF in the previous 3 years.^10^

Comorbidities and procedures were taken from the index and from any admissions in the year before the index, as described in our previous studies on readmissions in HF patients^10^ ^11^ (see online supplementary table A1). To investigate differences by patient characteristics, we defined two ‘extreme’ subgroups: a young group, aged <65, who had with fewer than three comorbidities from our list and an elderly multimorbid group, aged 80+, with at least three comorbidities from our list.

10.1136/bmjopen-2015-010669.supp1 Supplementary table List and descriptions of patient factors used in risk-adjustment regression models

### Measures of hospital use

We linked the index admissions to ED attendances and OPD appointments for up to 365 days after the discharge date of the index admission. Duplicate OPD appointments and those cancelled by the hospital were removed; for some analyses, we also dropped OPD appointments cancelled by the patient. The specialty was noted. Several mental health specialties were combined, and diabetic medicine was combined with endocrinology.

Subsequent admissions were divided into elective and emergency based on the ‘method of admission’ field and counted. It was noted via the ‘disposal’ field whether the ED attendances ended in admission. OPD non-attendance was flagged using the ‘attended’ field. For the time from discharge to first ED attendance and first OPD appointment, we ignored any intervening admission. In contrast, for readmission, we tracked forward in time to find the next admission for each patient. If that next admission was an emergency, it was counted as a readmission. If, however, the next one was an elective, then it was not counted as a readmission, which is the usual (strict) definition of a readmission.

### Analysis

Patient characteristics and hospital use were summarised for all patients who survived the index admission. For the tables, we simply present rates or other summaries; χ^2^ tests were used to compare proportions. For the plots of activity over time within the first year after discharge, we accounted for the competing risk of death using cumulative incidence rates.^12^ Kaplan-Meier curves treat deaths as censored, giving invalid risk estimates for non-death outcomes. As we had out-of-hospital deaths linked to the admissions database only for deaths up to August 2011, for these plots we used index admissions between April 2009 and August 2010, to allow a full year's follow-up. For OPD appointments within 2 weeks of discharge, we assumed that the proportion of patients discharged alive but who died within 2 weeks was negligible and so used the full set of patients.

### Patient involvement

Given our specific aims, no patients were involved in setting the research question or the outcome measures, and nor were they involved in the design and implementation of the study. We will work with colleagues at the National Institute for Health Research (NIHR) Imperial College Patient Safety Translational Research Centre to advise on plans for dissemination of these findings.

## Results

### All patients combined

There were 82 241 index admissions between April 2009 and March 2011, with 12 393 (15.1%) ending in death: 4.5% were aged under 65 with fewer than three comorbidities, of whom 5.4% died during the index HF admission, and 36.0% were aged 80+ with three or more comorbidities, of whom 21.5% died during the index HF admission. Patients were mostly elderly and multimorbid (table 1). All results below refer to the 69 848 survivors of the index admission.

**Table 1:** Characteristics of patients discharged alive from their index HF admission

Age group	Number	Per cent
18–44	812	1.2
45–64	7462	10.7
65–79	24 759	35.4
80+	36 815	52.7
Sex		
Male	34 988	50.1
Female	34 860	49.9
Age group: males		
Male 18–44	519	1.5
Male 45–64	5011	14.3
Male 65–79	14 158	40.5
Male 80+	15 300	43.7
Age group: females		
Female 18–44	293	0.8
Female 45–64	2451	7.0
Female 65–79	10 601	30.4
Female 80+	21 515	61.7
IMD quintile		
1 (least deprived)	10 260	14.7
2	13 668	19.6
3	14 933	21.4
4	15 638	22.4
5 (most deprived)	15 349	22.0
Living alone	6523	9.3
CABG	1008	1.4
PTCA	1913	2.7
CRT	287	0.4
Other pacing	2491	3.6
Stroke	1550	2.2
Pneumonia	8906	12.8
Ischaemic heart disease	33 966	48.6
Dementia	3387	4.8
Arrhythmias	39 902	57.1
Valvular disease	18 847	27.0
Peripheral vascular disease	6580	9.4
Hypertension	44 858	64.2
Chronic pulmonary disease	18 184	26.0
Diabetes mellitus	21 480	30.8
Renal disease	16 289	23.3
Obesity	3733	5.3
Mental health	6400	9.2
3+ comorbidities	45 164	64.7
3+ comorbidities other than hypertension	33 562	48.1
Arrhythmias and hypertension	26 032	37.3
IHD and hypertension	23 830	34.1
IHD and arrhythmias	19 741	28.3
Subgroup		
0 (neither of the below)	38 340	54.9
1=Young (<65 years old with <3 comorbidities)	3515	5.0
2=Elderly comorbid (aged 80+ with 3+ comorbidities)	23 234	33.3
Index LOS (nights)		
0–2	14 991	21.5
3–6	17 367	24.9
7–20	28 047	40.2
21+	9443	13.5

CABG, coronary artery bypass graft; CRT, cardiac resynchronisation therapy; IHD, ischaemic heart disease; IMD, Index of Multiple Deprivation; LOS, length of stay; PTCA, percutaneous transluminal coronary angioplasty.

ED attendances were common after the index admission. Of patients, 6.7% attended the ED within a week of index discharge, of which 77.6% resulted in readmission. Of ED attendances within the year, 70.5% resulted in admission. The 30-day all-cause readmission rate was 19.7%, whereas the 30-day rate for readmissions with HF as the primary diagnosis was only 5.6%. Just over half of all index survivors were readmitted as an emergency within a year: around a quarter of these had HF as the primary diagnosis. During the same period, about one in four patients had one or more elective admissions, totalling 36 481 elective admissions. Other than for cataracts, these were often diagnostic procedures or for cardiac pacing. Seventy-four per cent were same-day discharges.

Over 85% of patients were offered at least one OPD appointment in the year after the index discharge: by ‘offered’ we included all appointments not cancelled by the hospital, that is, including those not attended or cancelled by the patient. Cardiology was the most commonly used OPD specialty, with the anticoagulant service second most common (table 2). Overall, patients who were offered at least one appointment during the year were offered a median of six (table 3). One in ten patients saw three or more different specialties.

**Table 2:** Top 15 specialties for OPD appointments in year after index HF admission, ranked by total number of appointments

Specialty	Total number of appointments (% of total)	Number (%) patients with appointment	Ranking of specialties based on number of patients attending	% appointments not attended
Cardiology	113 398 (24.7)	34 702 (49.7)	1	11.9
Anticoagulant services	57 090 (12.5)	5489 (7.9)	8	8.2
Ophthalmology	32 657 (7.1)	13 618 (19.5)	2	13.4
General medicine	23 674 (5.2)	9647 (13.8)	3	10.4
Nephrology	23 182 (5.1)	5796 (8.3)	6	10.6
Clinical haematology	20 720 (4.5)	4905 (7.0)	12	8.7
Geriatric medicine	19 230 (4.2)	8393 (12.0)	4	14.0
Respiratory medicine	17 130 (3.7)	7985 (11.4)	5	14.0
Endocrinology	16 244 (3.5)	5216 (7.5)	9	12.7
Trauma and orthopaedics	12 538 (2.7)	5750 (8.2)	7	10.9
Urology	9921 (2.2)	4981 (7.1)	11	14.0
General surgery	9228 (2.0)	5163 (7.4)	10	11.4
Dermatology	8735 (1.9)	3544 (5.1)	14	9.9
Ear, nose and throat (ENT)	6507 (1.4)	3670 (5.3)	13	11.7
Gastroenterology	5353 (1.2)	3323 (4.8)	15	14.8

HF, heart failure; OPD, outpatient department.

**Table 3:** Hospital contacts in year after index admission overall and by the patient subgroup

	Patients aged <65 and not multimorbid	Patients aged 80+ and multimorbid	All patients
Emergency adms: % with none	58.6	34.5	40.3
Emergency adms: % with 1–2	33.0	48.3	44.1
Emergency adms: % with 3+	8.4	17.2	15.6
7-day emergency readmission rate	4.8	7.5	6.8
30-day emergency readmission rate	14.0	22.0	19.7
365-day emergency readmission rate	33.2	59.5	52.2
Elective adms: % with none	56.1	79.7	71.7
Elective adms: % with 1+	43.9	20.3	28.3
Median and IQR for inpatient bed days	1 (0 to 6)	4 (1 to 12)	3 (0 to 10)
OPD appts: % with none*	5.9	19.4	14.5
OPD appts: % with 1+*	94.1	80.6	85.5
% With any OPD appt within 2 weeks of discharge*	35.5	23.8	28.2
% With cardiology OPD appt within 2 weeks of discharge*	12.6	4.3	6.3
% Admitted on same day as attended OPD appt	1.0	0.6	0.7
ED attendances not ending in admission: % with none	76.4	76.4	76.1
ED attendances not ending in admission: % with 1+	23.6	23.6	23.9
% Who die within a year of index discharge	9.8	36.6	27.3

All comparisons between the young group and the elderly group have p<0.0001 except for ED attendances not ending in admission (p=0.99).

*Includes all OPD appointments irrespective of whether attended or not and includes those cancelled by the patient.

ED, emergency department; OPD, outpatient department.

For all patients, there was a median of 27 days between discharge and the first appointment. Of these, 9.7% were cancelled by the patient, 12.9% were missed by the patient on the day and 1.9% resulted in admission on the same day. Furthermore, 30.1% were at cardiology clinics, with ophthalmology and Medicine for the Elderly (geriatric medicine) being the two next commonest. Only 6.8% of patients were offered a cardiology appointment within 2 weeks; for all specialties combined, the proportion reviewed within 2 weeks was 28.2%.

### Results by the patient subgroup

In the young subgroup, 5.1% had an ED attendance within a week of discharge, of whom 64.9% were admitted. For the elderly multimorbid subgroup, these figures were significantly higher at 7.3% and 80.0% (both p<0.001). Within a year of discharge, 43.2% in the young subgroup had an ED attendance, of whom only 58.5% were admitted. For the elderly multimorbid subgroup, these figures were again significantly higher at 61.9% and 73.7% (both p<0.001). The time to first attendance was similar for both groups.

Readmission rates were consistently higher in the elderly multimorbid patients than in young patients, and the primary diagnosis differed little by age. Of the 30-day readmissions, 30.4% in the young patients and 28.1% in the elderly multimorbid patients were for HF (p=0.268); of the 365-day readmissions, 24.5% in the young patients and just 22.3% in the elderly multimorbid patients were for HF (p=0.079). Elective admission rates were twice as common for the young patients than for the elderly multimorbid patients. The total number of inpatient bed days in the year after index discharge was 91 254 in the young group and 357 554 in the elderly group, with 943 745 bed days for all patients combined.

**Figure 1:**
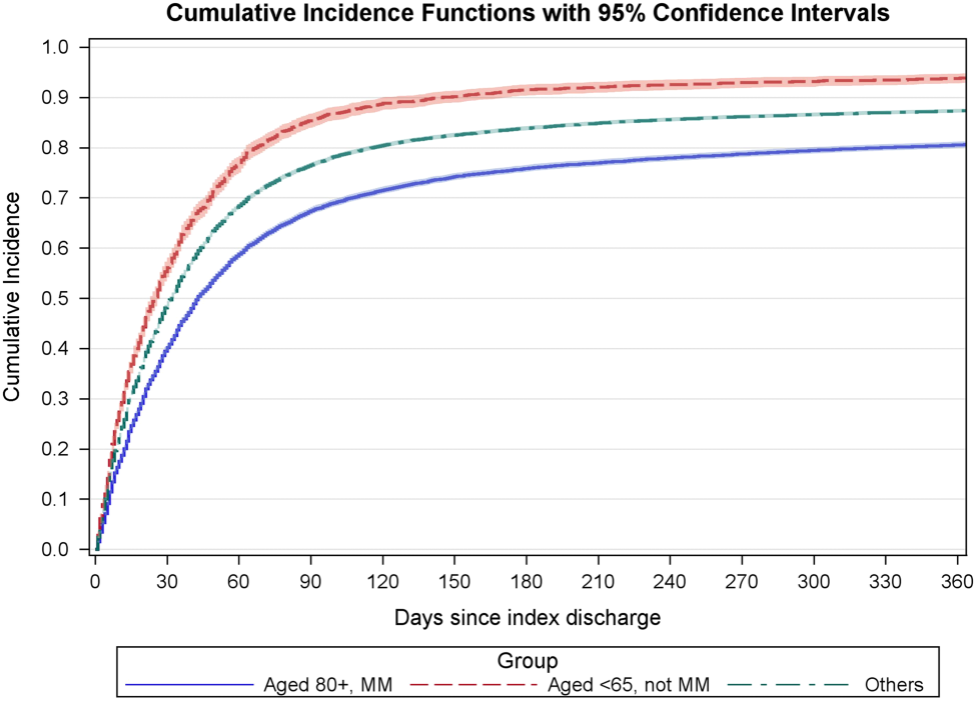
Cumulative proportion of patients with at least one outpatient department (OPD) appointment (any specialty) by number of days since index discharge. MM, multimorbid.

For both subgroups, outpatient appointments were common in the year after the index HF admission. Using cumulative incidences, only about 5% of patients in the young subgroup had no outpatient appointments, compared with about 20% of patients in the elderly multimorbid subgroup (figure 1, table 3). Young patients with at least one outpatient appointment had on average more than double the number of appointments compared with the equivalent elderly group. Young patients were seen 10 days earlier on average (median 20 days since index discharge compared with 30 days).

Regarding the NICE guideline, 12.6% of the young group and 4.3% of the elderly multimorbid group were offered a cardiology outpatient appointment within 2 weeks of discharge; OPD follow-up rates for all specialties combined at 2 weeks were 35.5% for the young group and 23.8% for the elderly multimorbid group. After cardiology, the next most common specialty for both subgroups was the anticoagulant service, followed by clinical haematology and general medicine in the young group and ophthalmology and Medicine for the Elderly (1.7% of patients) in the elderly multimorbid group.

**Figure 2:**
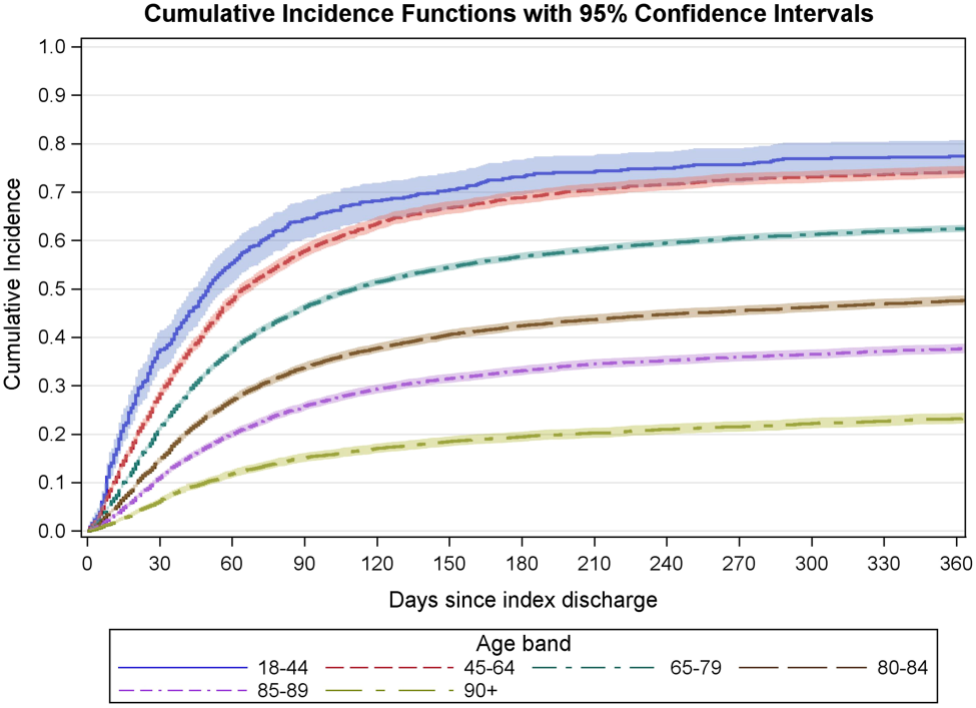
Cumulative proportion of patients offered at least one cardiology outpatient department (OPD) appointment in the year following the index heart failure (HF) admission by the age group.

As the cardiology OPD, follow-up rates differed greatly between our young group and elderly multimorbid subgroup, we stratified by age and number of comorbidities. After stratifying just by age, the cardiology follow-up rates ranged from 2.4% in those aged 90+ to 19.6% in those aged under 45, an eightfold difference (figure 2). These age differences were statistically significant (p<0.0001, Gray's test for separation between the curves^13^).

**Figure 3:**
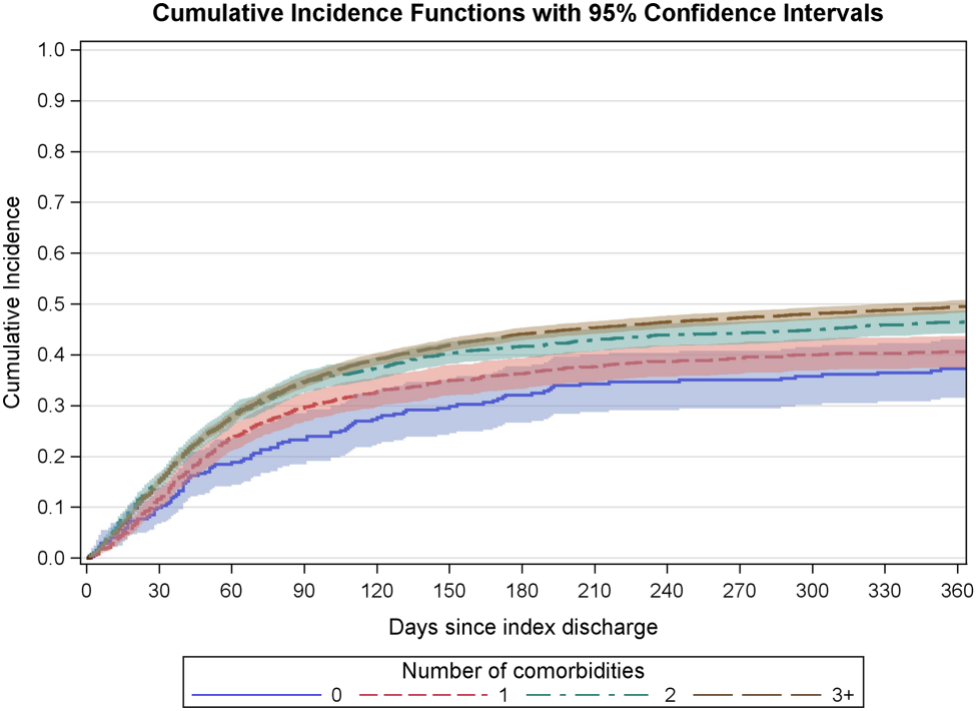
Cumulative proportion of patients aged 80–84 offered at least one cardiology OPD appointment in the year following the index HF admission by number of comorbidities.

Within each age group, the more comorbidities a patient had, the higher their cardiology follow-up rate. Figure 3 shows this for the commonest age group, 80–84, although it was true for all age bands. At the 2-week point and also throughout the year, the variation by age was greater than the variation by comorbidity.

## Discussion

### Summary of findings

In this national study of adult patients with an index admission for HF in England, the patients commonly had subsequent planned and unplanned hospital contact including inpatient, ED and OPD activity. ED reattendance was common: 6.7% within a week and 57.4% in a year—only one in four who attended in the year after discharge was not readmitted. Over half of all patients had at least one unplanned admission within a year, most being for non-HF diagnoses. OPD attendance was high and covered a broad range of specialties. Cardiology and anticoagulation services were the two most common, with low use by Medicine for the Elderly—surprising, given the age of population.

The proportion reviewed by a cardiologist in the OPD within 2 weeks of inpatient discharge as now recommended by NICE and American Heart Association guidelines was only 6.8%, with large differences by the patient subgroup. The proportion reviewed was highest for the youngest patients, an advantage maintained throughout the subsequent year. Patients with more comorbidities had a higher chance of cardiology follow-up, suggesting that the specialists take on more of the more complex cases. However, the effect of age appeared greater than that of comorbidity and operated in the opposite direction. Elderly patients did not seem to be seen more by other specialties instead including care of the elderly services—figure 1 shows that their follow-up rate in OPD for all specialties combined was still lower than that for patients under 65. The proportion of elderly multimorbid patients attending a general medicine clinic within 2 weeks of discharge was 1.5% (compared with 2.6% in the young subgroup), and the proportion for Medicine for the Elderly was only 1.7%.

### Results in relation to other studies

There are few national studies of hospital utilisation by patients with HF beyond those of readmissions. A study of geographical variation in hospital use in the healthcare system of the Department of Veterans Affairs for various chronic diseases including HF found large variations in inpatient but not OPD use across the USA,^14^ although a Medicare-GWTG study of 225 participating hospitals found that the early follow-up rate for HF ranged from under 10% to 64% by hospital.^6^ The ESC HF pilot registry covering participating centres in 12 European countries only reported mortality and readmission as outcomes.^2^ Ours is the first UK study to describe ED and OPD activity. As expected, use of cardiology and the anticoagulant service were common, but we were a little surprised by the large number of appointments for ophthalmology, which varied by age. An increasing amount of eye treatments such as laser photocoagulation now take place in the OPD. The large numbers of older people attending ophthalmology OPDs are added to by large numbers of individuals with diabetes with or at risk of eye disease,^15^ and nearly one in three of our cohort had diabetes recorded.

### Strengths and weakness of this study

England and the other UK countries benefit from national hospital databases like HES, enabling transfers and readmissions to any other NHS hospital to be tracked. With a time lag, records are also linked to death registrations, which we used to account for the competing risk of death in the cumulative incidence plots. As well as the large sample size, using national administrative data avoids the selection bias of clinical trials.

Administrative data also have limitations. A systematic review of studies of the data quality of HES found that for inpatient records the primary diagnosis was correct 96% of the time for studies since 2005.^16^ The accuracy of recording of secondary diagnoses varies by hospital, and the comorbidity frequencies that we calculated are likely to be underestimates despite tracking back to obtain information from previous admissions. Much less is known about ED and OPD records. HES ED counts have until recently regularly been compared against the number of recorded ED attendances in quarterly monitoring (QMAE) returns. In 2010–2011, there were 15.8 million attendances reported in ED HES (excluding planned follow-up appointments) compared with 21.4 million reported in QMAE.^17^ However, non-submitting walk-in centres and minor injury units account for the vast majority of the shortfall. The paucity of diagnosis information in ED and OPD records meant that we were unable to estimate reliably which attendances or appointments are primarily for the HF and which are for other problems.

As HES is a hospital database, it lacks information on activity in primary care, the sector in which much management and monitoring of patients take place. Patients with HF have on average 11–13 contacts per year with their GP or other members of the primary care team.^1^ Work from Spain suggests that there is also considerable variability in the use of those services and in the management of HF patients by GPs.^18^ HF is considered to be an ambulatory or primary care sensitive condition, one for which hospital admission could be prevented by interventions in primary care. However, practice-level quality of care scores did not correlate with the fall in admission rates in England.^19^ As HES would not capture primary care activity, any NICE-recommended initial postinpatient discharge follow-up by the specialist HF team that does occur in primary care would be missed. However, in the UK, this is uncommon and should not invalidate our use of OPD records.

Our use of an index HF admission simplifies what we intended as a simply descriptive analysis and represents a convenient reference point in time to examine service use.^20–22^ As this was their first emergency HF admission for at least 3 years, we assumed that either they had been stable during that time or they were new HF patients. A more sophisticated approach using multistate models, for example, could investigate the interrelations between the different NHS contacts.

HES data do not include diagnosis dates or information on where the diagnosis was made. A Canadian study found that half of HF patients have it diagnosed in the ED (14%) or as an inpatient (37%), with the other half mostly in general rather than specialist outpatient clinics;^23^ outcomes differed markedly depending on the place of diagnosis.

Lastly, we were restricted to 2009–2010 and 2010–2011 data owing to the unavailability of linked mortality files for more recent years. It will be interesting to repeat this analysis once sufficient data have accrued after the October 2014 NICE guidelines that recommend 2-week postdischarge cardiology follow-up.

### Implications for clinical practice

Our findings reaffirm the high mortality and high hospital service utilisation for ED, inpatient and outpatient sectors in patients with HF. The England and Wales national audit report, our recent study that combined the audit results with HES^7^ and work from outside the UK^6^ ^24^ all showed the benefit of cardiologist input and follow-up. This is now recommended by NICE and the ESC,^8^ ^25^ but UK guidelines have changed over time. We studied index admissions from April 2009 to March 2011. At the start of the study period, the guidance to the NHS in England from NICE (issued in July 2003)^26^ stated that “patients with heart failure should generally be discharged from hospital only when their clinical condition is stable and the management plan is optimised. Timing of discharge should take into account patient and carer wishes and the level of care and support that can be provided in the community.” There was no mention of care after discharge other than to state that all patients “require monitoring… to include clinical assessment, a review of medication, and serum urea, electrolytes, creatinine and eGFR,” with a recommendation that this should take place at least every 6 months. An update to this guidance was issued during our study period (on 25 August 2010),^27^ which reiterated the same advice but added a recommendation that during hospital admission the medical team should seek advice from a specialist in HF. Quality Standards related to this guidance were not issued until after our study period (June 2011).^28^

The lack of detailed advice on hospital and transitional care was recognised, and NICE issued new guidelines on the diagnosis and treatment of acute (ie, hospitalised) HF in 2014^8^ and related Quality Standards in 2015^29^ after the period of our study. Four recommendations related to the organisation of hospital and transitional care.^8^ All hospitals were to provide a specialist HF team based on a cardiology ward and providing outreach services; everyone admitted with suspected HF was to have early and continuing input from the specialist team; discharge from hospital and subsequent management in primary care (including ongoing monitoring and care by a multidisciplinary team) should be planned and a follow-up clinical assessment should be undertaken by a member of the specialist HF team within 2 weeks of the person being discharged from hospital. These recommendations were then endorsed in the new NHS Quality Standards for Acute Heart Failure, issued on 3 December 2015.^29^ It remains to be seen how quickly and consistently these standards will be implemented across the NHS in England. For our study period, only 6.7% of index survivors had a cardiology OPD appointment within 2 weeks of their index HF admission, and only half had one within a year. Our work suggests that there will need to be considerable organisational change to reach the 2-week follow-up target. It will be important to monitor progress against this measure in the future.

## Conclusions

Our results confirm that patients with HF often have multiple comorbidities and hence complex medical needs, with a high use of hospital services beyond the index acute admission. Our subgroup analyses by age and comorbidity show notable differences, which need to be addressed to meet best practice and the recent NICE and European guidance for specialist outpatient follow-up.
