# Supplementary material for: Use of hospital services by age and comorbidity after an index heart failure admission in England: an observational study
Source: BMJ Open. 2016 Jun 9;6(6):e010669. doi: 10.1136/bmjopen-2015-010669 (PMC4908910; doi:10.1136/bmjopen-2015-010669)
Supplement: Supplementary table — List and descriptions of patient factors used in risk-adjustment regression models [file bmjopen-2015-010669supp_table.pdf]

Supplementary Table A1. List and descriptions of patient factors used in risk-adjustment regression models

Factor	Codes and/or description (ICD10 unless specified)
Age	Categorised at 18-44 then five-year bands from 45-49 to 85-89, then 90+
Sex	
Carstairs deprivation fifth	Measure of socio-economic status of small geographical area of residence
CABG in year before or during index HF admission	OPCS codes K40, K41, K42, K43, K44, K45, K46
PTCA in year before or during index HF admission	OPCS codes K49, K50, K75
Implantable cardioverter defibrillator implanted in year before or during index HF admission	OPCS codes K590, K591, K592, K593, K594, K596, K598, K599
Pacemaker (not CRT) inserted in year before or during index HF admission	OPCS codes K600, K601, K602, K603, K605, K606, K607, K608, K609, K610, K611,K612, K613, K615, K616, K617, K618, K619
CRT inserted in year before or during index HF admission	OPCS codes K607, K617
OPD appointments missed in year before index HF admission	

OPD appointments attended in year before index HF admission	
Comorbidity flags, all defined using ICD10 codes:	All comorbidity flags were based on information during the index admission or in any admission in previous year
Stroke	I60, I61, I62, I63, I64
Pneumonia	J12, J13, J14, J15, J16, J17, J18
Ischaemic heart disease	I20, I21, I22, I23, I25
Dementia	F00, F01, F02, F051
Arrhythmias	I441, I442, I443, I456, I459, I47, I48, I49, R000, R001, R008, T821, Z450, Z950
Heart valve disorders	A520, I05, I06, I07, I08, I091, I098, I34, I35, I36, I37, I38, I39, Q230, Q231, Q232, Q233, Z952, Z953, Z954
Peripheral vascular disease	I70, I71, I731, I738, I739, I771, I790, I792, K551, K558, K559, Z958, Z959
Hypertension	I10, I11, I12, I13, I15
Chronic lung diseases	I278, I279, J40, J41, J42, J43, J44, J45, J46, J47, J60, J61, J62, J63, J64, J65, J66, J67, J684, J701, J703
Diabetes	E10, E11, E12, E13, E14
Renal disease	I120, I131, N18, N19, N250, Z490, Z491, Z492, Z940, Z992

Obesity	E66
Any mental health condition (except dementia)	F06, F07, F09, then rest of F chapter from F20 onwards
Living alone	Z602
